# Correction: Culprit Vessel Only versus Multivessel Percutaneous Coronary Intervention in Patients Presenting with ST-Segment Elevation Myocardial Infarction and Multivessel Disease

**DOI:** 10.1371/journal.pone.0101073

**Published:** 2014-06-24

**Authors:** The PLOS ONE Staff

## Notice of Republication

This article was republished on May 29, 2014, due to figure errors. Please download this article again to view the correct version. The originally published, uncorrected article and the republished, corrected article are provided here for reference.

## Supporting Information

File S1 Originally published, uncorrected article.
(PDF)

File S2 Republished, corrected article.
(PDF)
