# Correction: Culprit Vessel Only versus Multivessel Percutaneous Coronary Intervention in Patients Presenting with ST-Segment Elevation Myocardial Infarction and Multivessel Disease

**DOI:** 10.1371/journal.pone.0116450

**Published:** 2014-12-19

**Authors:** The PLOS ONE Staff

The affiliation for all authors is incorrect. The affiliation for all authors should read: Department of Cardiology, Beijing An Zhen Hospital, Capital Medical University, Beijing Institute of Heart, Lung and Blood Vessel Disease, Beijing, China.
