# Supplementary material for: Correction: Culprit Vessel Only versus Multivessel Percutaneous Coronary Intervention in Patients Presenting with ST-Segment Elevation Myocardial Infarction and Multivessel Disease
Source: PLoS One. 2014 Jun 24;9(6):e101073. doi: 10.1371/journal.pone.0101073 (PMC4069192; doi:10.1371/journal.pone.0101073)
Supplement: File S2 — Republished, corrected article. (PDF) [file pone.0101073.s002.pdf]

OPEN ACCESS Freely available online

PLOS ONE

Culprit Vessel Only versus Multivessel Percutaneous Coronary Intervention in Patients Presenting with ST-Segment Elevation Myocardial Infarction and Multivessel Disease

Dongfeng Zhang, Xiantao Song*, Shuzheng Lv, Fei Yuan, Feng Xu, Min Zhang, Wei Li, Shuai Yan

Department of Cardiology, Capital Medical University affiliated Beijing Anzhen Hospital, Beijing Institute of Heart, Lung and Blood Vessel Disease, Beijing, China

Abstract

Background: The best strategy for ST-segment elevation myocardial infarction (STEMI) patients with multivessel disease (MVD), who underwent primary percutaneous coronary intervention (PCI) in the acute phase, is not well established.

Objectives: Our goal was to conduct a meta-analysis comparing culprit vessel only percutaneous coronary intervention (culprit PCI) with multivessel percutaneous coronary intervention (MV-PCI) for treatment of patients with STEMI and MVD.

Methods: Pubmed, Elsevier, Embase, and China National Knowledge Infrastructure (CNKI) databases were systematically searched for randomized and nonrandomized studies comparing culprit PCI and MV-PCI strategies during the index procedure. A meta-analysis was performed using Review Manager 5.1 (Cochrane Center, Denmark).

Results: Four randomized and fourteen nonrandomized studies involving 39,390 patients were included. MV-PCI strategy is associated with an increased short-term mortality (OR: 0.50, 95% CI: 0.32 to 0.77, $p = 0.002$), long-term mortality (OR: 0.52, 95% CI: 0.36 to 0.74, $p < 0.001$), and risk of renal dysfunction (OR: 0.77, 95% CI: 0.61 to 0.97, $p = 0.03$) compared with culprit PCI strategy, while it reduced the incidence of revascularization (OR: 2.65, 95% CI: 1.80 to 3.90, $p < 0.001$).

Conclusions: This meta-analysis supports current guidelines which indicate that the non-culprit vessel should not be treated during the index procedure.

Citation: Zhang D, Song X, Lv S, Yuan F, Xu F, et al. (2014) Culprit Vessel Only versus Multivessel Percutaneous Coronary Intervention in Patients Presenting with ST-Segment Elevation Myocardial Infarction and Multivessel Disease. PLoS ONE 9(3): e92316. doi:10.1371/journal.pone.0092316

Editor: Claudio Moretti, S.G.Battista Hospital, Italy

Received October 30, 2013; **Accepted** February 21, 2014; **Published** March 20, 2014

Copyright: © 2014 Zhang et al. This is an open-access article distributed under the terms of the Creative Commons Attribution License, which permits unrestricted use, distribution, and reproduction in any medium, provided the original author and source are credited.

Funding: State Science and Technology Support Program (No. 2011BAI11B05) http://program.most.gov.cn/. The funders had no role in study design, data collection and analysis, decision to publish, or preparation of the manuscript.

Competing Interests: The authors have declared that no competing interests exist.

* E-mail: xiantao_song@163.com

Introduction

Acute ST-segment elevation myocardial infarction (STEMI) is a huge public health burden that affects many people worldwide every year. Approximately 40% to 65% of the patients presenting with STEMI have multivessel disease (MVD), which is associated with worse clinical outcomes than single-vessel disease (SVD) [1]. Percutaneous coronary intervention (PCI) is currently the favorable reperfusion treatment of choice in patients with STEMI. However, optimal strategies for STEMI patients with MVD during the index procedure, whether to treat non-culprit vessels, are still unclear.

2012 ESC guidelines [2] recommend that primary PCI should be limited to the culprit vessel with the exception of cardiogenic shock and persistent ischemia after PCI of the supposed culprit lesion while 2011 ACCF/AHA/SCAI PCI guidelines [3] suggest that PCI should not be performed in a non-culprit vessel at the time of primary PCI in patients with STEMI without hemodynamic compromise, where the classes and levels of evidence are IIaB and IIIB respectively. However, these suggestions were based on some retrospective or small observational studies which did not have high evidence level. The main factors supporting these guidelines are summarized as follows: complications related to non-culprit vessel PCI, overvalued stenosis, renal insufficiency, and low success rates. The advancements in PCI technology and adjunctive pharmacotherapy have led some interventionalists to operate outside of established guidelines.

Several researches showed inconsistent outcomes. Our goal was to compare the safety and efficacy of culprit vessel only PCI (culprit PCI) and multivessel PCI (MV-PCI) during the index procedure in patients with STEMI and MVD quantitatively. Therefore, we conducted a meta-analysis of randomized and nonrandomized studies.

Methods

Search Strategy

Pubmed, Elsevier, Embase, and China National Knowledge Infrastructure (CNKI) databases were systematically searched by two independent investigators (S.Y and W.L) for all articles

Table 1. Main Characteristics of Included Studies.

	Primary Author	Year Published	Setting	Symptom Time, h	PCI strategies		Maximum Follow-Up
					Culprit PCI	MV-PCI	
Randomized studies							
1	Di Mario	2004	Multicenter	12	17	52	1 yr
2	Ochala	2004	Single-center	12	44	48	6 months
3	Politi	2010	Single-center	12	84	65	2.5±1.4 yrs
4	Wald	2013	Multicenter	–	231	234	23 months
Nonrandomized studies							
5	Abe	2013	Multicenter	12	220	54	1 yr
6	Bauer	2013	Multicenter	–	2118	419	In hospital
7	Carvender	2009	Multicenter	All	25802	3134	In hospital
8	Corpus	2004	Single-center	12	354	26	1 yr
9	Dziewierz	2010	Multicenter	–	707	70	1 yr
10	Hannan	2010	Multicenter	24	503	503	3.5 yrs
11	Jensen	2012	Multicenter	12	820	354	2 yrs
12	Khattab	2008	Single-center	12	45	28	1 yr
13	kornowski	2011	Multicenter	12	393	275	3 yrs
14	Mohamad	2011	Single-center	12	30	7	1 yr
15	Qarawani	2008	Single-center	12	25	95	1 yr
16	Roe	2001	Multicenter	–	61	68	6 months
17	Toma	2010	Multicenter	6	1984	217	3 months
18	Varani	2008	Single-center	24	156	147	1.7±1.0 yrs
Total					33594	5796	

doi:10.1371/journal.pone.0092316.t001

published before 6 October 2013. The following keywords were used for the search: "percutaneous coronary intervention", "ST-segment elevation myocardial infarction", and "multivessel disease". Studies were excluded if they met any one of the following criteria: (1) duplicate publication, (2) ongoing or unpublished study, and (3) publication only as an abstract or as conference proceedings. References of retrieved studies were searched manually for additional potentially relevant articles. Authors of studies were contacted when results were unclear or when relevant data were not reported. Differences in investigator assessments of articles were resolved by discussing with a third investigator (D.F.Z). No language restrictions were enforced.

Study Selection

An initial screening of titles or abstracts was conducted, followed by full-text reviews. Studies' eligibility criteria included the followings: 1) a study population of STEMI patients with MVD; 2) PCI procedures included both culprit PCI and MV-PCI; 3) MV-PCI was performed during the index procedure; and 4) studies that reported quality assessment, data extraction, and endpoint data of interest. Randomized and nonrandomized studies were included. Exclusion criteria were: patient populations without concurrent STEMI and MVD, comparisons without culprit PCI or MV-PCI, and MV-PCI performed after the index procedure. Reviews, editorials, meeting abstracts, and commentaries were excluded from our analysis.

Quality Assessment

The quality of randomized studies was assessed using methods recommended by the Cochrane Collaboration based on the following six components: 1) sequence generation for allocation; 2) allocation concealment; 3) blinding of participants, personnel, and outcome assessors; 4) incomplete outcome data; 5) selective outcome reporting; and 6) other sources of bias. For nonrandomized studies, quality was assessed based on control of confounders, blinded assessment of angiography data, and preferred PCI strategy.

Data Extraction

Data were abstracted on prespecified forms by two reviewers (W.L and S.Y) that were not involved in any of the studies retrieved. Divergent assessments were resolved by discussing with a third investigator (D.F.Z). Study information was recorded as follows: study design, quality indicators, baseline clinical characteristics, and clinical outcomes.

Definition and Endpoints

The culprit PCI strategy was defined as PCI confined to culprit vessel lesions only. The MV-PCI strategy was defined as PCI in which lesions in the culprit vessel as well as ≥1 nonculprit vessel lesions. All the interventions should have had taken place within the index procedure. MVD was defined as reported in each study. The primary endpoints were short-term (in hospital/30 days) and long-term mortality. Secondary endpoints included rates of renal dysfunction, reinfarction, and revascularization. Renal dysfunction as well as reinfarction and revascularization were defined as reported in each study. Mortality included both cardiac and no cardiac death.

Statistical Analysis

All statistical analysis was performed using Review Manager 5.1 (Cochrane Center, Denmark). Odds ratio (OR) and 95% confidence intervals (95% CI) were used as summary statistics. Heterogeneity across studies was analyzed using I^2 [$I^2 = (Q\text{-df})/Q$; where Q is the chi-square statistic and df is the degree of freedom]. Values of $I^2 > 50\%$ were considered statistically significant. Pooled estimates were first calculated using the Mantel-Haenszel fixed-effects model, whereas the DerSimonian and Lair random-effects model was used if there was heterogeneity.

The following methods were used to explore sources of heterogeneity: (1) subgroup analysis (randomized and nonrandomized studies); and (2) sensitivity analysis performed by excluding trials which potentially biased meta-analysis results.

Potential publication bias was examined by visual inspection of a funnel plot. All p values were 2-tailed, with statistical significance set at $p < 0.05$. This study was performed according to the MOOSE (Meta-Analysis of Observational Studies in Epidemiology) [4] statement.

Results

Eighteen studies including 39,390 patients comparing culprit PCI versus MV-PCI in patients with STEMI and MVD during the index procedure were identified finally (Table 1), four randomized [5,6,7,8] and fourteen nonrandomized studies [9,10,11,12,13,14,15,16,17,18,19,20,21,22] published between 2001 and 2013. Nine of the fourteen nonrandomized studies were subanalyses of prospective registries. Details of the screening process for eligible studies are shown in Fig. 1. Quality assessment results are detailed in Table 2 and Table 3.

Study Characteristics

Culprit PCI was the more frequently performed PCI strategy (33,594 of 39,390 patients, 85.3%). Baseline characteristics of the included studies are presented in Table 4. Compared with the culprit PCI group, patients in the MV-PCI group had a lower rate of diabetes, hypertension and hyperlipidemia. Six studies excluded the patients with cardiogenic shock [5,6,7,8,14,19], and three studies reported the rate of cardiogenic shock [11,16,21]. Ten studies gave information of the use of GP IIb/IIIa inhibitors.

Table 2. Quality of Randomized Studies.

Primary author	Adequate sequence generation of allocation	Allocation concealment	Blinding of participants, personnel, and outcome assessors	Complete outcome data	Free of selective outcome reporting	Free of other sources of bias
Di Mario	Unclear	Unclear	Unclear	Yes	Unclear	Unclear
Ochala	Unclear	Unclear	Unclear	Yes	Unclear	Unclear
Politi	Yes	Unclear	Unclear	Yes	Unclear	Unclear
Wald	Yes	Unclear	Yes	No	Yes	Unclear

doi:10.1371/journal.pone.0092316.t002

Table 3. Quality of nonrandomized Studies.

Primary author	Control of confounders	Blinded assessment of angiography data	Preferred PCI strategy
Abe	±(subanalysis of prospective registry)	–	Operator decision
Bauer	±(subanalysis of prospective registry)	–	–
Carvender	±(subanalysis of prospective registry)	–	–
Corpus	–	–	Operator decision
Dziewierz	±(subanalysis of prospective registry)	–	–
Hannan	±(subanalysis of prospective registry)	–	–
Jensen	±(subanalysis of prospective registry)	–	Operator decision
Khattab	Prospective observational	–	Operator decision
Kornowski	±(subanalysis of prospective registry)	–	Operator decision
Mohamad	–	–	–
Qarawani	–	–	Operator decision
Roe	–	–	Operator decision
Toma	±(subanalysis of prospective registry)	–	Operator decision
Varani	±(subanalysis of prospective registry)	–	Operator decision

doi:10.1371/journal.pone.0092316.t003

Main Outcomes

Short-term mortality was reported in 15 studies including 36,687 patients. In-hospital or 30-day death occurred in 1,515 of 31,349 patients (4.83%) who underwent culprit PCI versus 370 of 5,338 patients (6.93%) who received MV-PCI. Signs of heterogeneity were found across trials ($I^2 = 70\%$) and a randomized model was used. Compared with culprit PCI, MV-PCI was associated with an increased short-term mortality (OR: 0.50, 95% CI: 0.32 to 0.77, p = 0.002). Pooled short-term outcome data are detailed in Fig. 2.

Long-term mortality for both strategies was reported in 16 studies including 7,905 patients. There were 362 long-term follow up deaths among 5,670 patients (6.38%) who received culprit PCI, whereas 245 deaths occurred among 2,235 (10.96%) patients who received MV-PCI. Heterogeneity was found across trials ($I^2 = 67\%$) and a randomized model was used. MV-PCI was associated with an obviously increased long-term mortality in comparison with culprit PCI strategy (OR: 0.52, 95% CI: 0.36 to 0.74, p<0.001). Pooled long-term outcome data are illustrated in Fig. 3.

Four studies are available of the short-term renal dysfunction detail. Politi et al. [7] defined renal dysfunction as an increase in serum creatinine values of 0.5 mg/dl or greater or a 25% or greater relative increase from baseline within 72 hours following PCI. Abe et al. [9] defined renal dysfunction as an increase in serum creatinine values of 0.5 mg/dl or greater or a 25% or greater relative increase from baseline within 1 week following exposure to contrast medium. Cavender et al. [11] defined renal dysfunction as a new requirement for dialysis or an increase in creatinine to >2 mg/dl and 2 times the baseline creatinine. Qarawani et al. [19] defined it as a rise of 30% and more in creatinine within 24 hours from the baseline value. To sum up, renal dysfunction occurred in 503 of 26,131 patients (1.92%) who underwent culprit PCI versus 93 of 3,348 patients (2.78%) who received MV-PCI. No heterogeneity was found among the studies ($I^2 = 0\%$) and a fixed effects model was used. The difference between two groups are significant (OR: 0.77, 95% CI: 0.61 to 0.97, p = 0.03), which indicates that the MV-PCI may increase the risk of renal dysfunction because of the high dose of contrast agent (Fig. 4).

Nine articles reported on long-term reinfarction, 1,449 cases in the culprit PCI group and 847 cases in the MV-PCI group. No heterogeneity was found among the studies ($I^2 = 41\%$) and a fixed effects model was used. No significant difference was found between the two groups (OR: 1.13, 95% CI: 0.76 to 1.67, p = 0.55) (Fig. 5).

Five studies gave the information of long-term revascularization, 421 cases in the culprit PCI group and 424 cases in the MV-PCI group. Signs of heterogeneity were not found across trials ($I^2 = 46\%$) and a fixed model was used. MV-PCI was associated with an obviously decreased long-term revascularization in comparison with culprit PCI strategy (OR: 2.65, 95% CI: 1.80 to 3.90, p<0.001) (Fig. 6).

Sensitivity Analysis

We performed sensitivity analyses by repeating analyses following removal of each study, one at a time (data not shown). No single study had excessive influence on the results for primary or secondary endpoints.

The results of randomized trials only and both randomized and nonrandomized trials are different which showed in Table 5. Assessment of funnel plots suggested no publication bias.

Discussion

Our analysis suggested that MV-PCI strategy is associated with an increased short-term mortality (OR: 0.50, 95% CI: 0.32 to 0.77, p = 0.002), long-term mortality (OR: 0.52, 95% CI: 0.36 to 0.74, p<0.001), and risk of renal dysfunction (OR: 0.77, 95% CI: 0.61 to 0.97, p = 0.03) compared with culprit PCI strategy, while it reduced the incidence of revascularization (OR: 2.65, 95% CI: 1.80 to 3.90, p<0.001). No significant difference was found between the two groups in terms of the rate of reinfarction.

MVD has been proved to be associated with a poor prognosis in STEMI patients. Appropriate management of these patients has always been a topic of debate. Current guidelines recommend that in the absence of hemodynamic compromise, PCI during STEMI should only focus on the culprit lesion. Other lesions are addressed during subsequent elective revascularization. Justifications for these guidelines include [23]: 1) the acute phase of STEMI is a

Table 4. Baseline Characteristics of Included Studies.

	Primary Author	Year Published	GP IIb/IIIa, %		Age, mean, yrs		Male, %		Diabetes, %		Hypertension, %		Hyperlipidemia, %		Shock, %		Smoker, %	
			Culprit PCI	MV-PCI	Culprit PCI	MV-PCI	Culprit PCI	MV-PCI	Culprit PCI	MV-PCI	Culprit PCI	MV-PCI	Culprit PCI	MV-PCI	Culprit PCI	MV-PCI	Culprit PCI	MV-PCI
Randomized studies																		
1	Di Mario	2004	82.4	75	65.3±7.4	63.5±12.4	84.6	88.2	41.2	11.5	58.8	36.5	52.9	41.2	excl	excl	81	66.6
2	Ochala	2004	50.7	51.1	67±7.9	65±8.3	75	72.9	34.1	31.2	47.7	52.1	90.9	81.2	excl	excl	43.1	37.5
3	Politi	2010	100	100	66.5±13.2	64.5±11.7	76.2	76.9	23.8	13.8	59.5	49.2	N/A	N/A	excl	excl	N/A	N/A
4	Wald	2013	76	76	62(33–90)*	62(32–92)*	81	76	21	15	40	40	N/A	N/A	excl	excl	45	50
Nonrandomized studies																		
5	Abe	2013	N/A	N/A	68.6±11.7	72.0±11.7	77.3	77.8	43.2	50	65.9	62.9	52.7	48.1	N/A	N/A	60	57.4
6	Bauer	2011	48.9	60.2	65±12.2	62.8±12	73.8	76.1	23.1	22.4	60.7	62.4	44.6	52.5	N/A	N/A	55.4	55
7	Carvender	2009	N/A	N/A	62(53–73)*	60(52–72)*	72.1	71.5	23.4	24.7	63.2	60.4	58.6	56.5	10.3	13.8	64.8	63.2
8	Corpus	2004	N/A	N/A	N/A	N/A	N/A	N/A	N/A	N/A	N/A	N/A	N/A	N/A	N/A	N/A	N/A	N/A
9	Dziewierz	2010	N/A	N/A	N/A	N/A	N/A	N/A	N/A	N/A	N/A	N/A	N/A	N/A	N/A	N/A	N/A	N/A
10	Hannan	2010	N/A	N/A	N/A	N/A	78.7	75	21.4	23.7	N/A	N/A	N/A	N/A	excl	excl	N/A	N/A
11	Jensen	2013	79	61.3	N/A	N/A	79.8	76	10.1	10.7	27.4	21.5	N/A	N/A	N/A	N/A	62.4	46.9
12	Khattab	2008	44	36	65±13	69±12	78	75	16	7	82	75	80	79	4.4	3.6	40	36
13	Kornowski	2011	54.6	54.5	63.5	62	80.9	79.6	18.1	15.3	57.5	54.9	41.7	48	N/A	N/A	62.8	61.3
14	Mohamad	2011	N/A	N/A	N/A	N/A	N/A	N/A	N/A	N/A	N/A	N/A	N/A	N/A	N/A	N/A	N/A	N/A
15	Qarawani	2008	96	94.7	67±3.7	66±3.2	64	65	16	12.6	40	37.8	16	13.6	excl	excl	60	61
16	Roe	2001	N/A	N/A	N/A	N/A	N/A	N/A	N/A	N/A	N/A	N/A	N/A	N/A	N/A	N/A	N/A	N/A
17	Toma	2010	71.9	78.8	64(55–73)*	64(53–74)*	79.4	77.4	20	11.5	55.6	47.5	N/A	N/A	1.2	1.8	39.9	38.2
18	Varani	2008	N/A	N/A	69.8±13	68.7±13	75	67	N/A	N/A	N/A	N/A	N/A	N/A	N/A	N/A	N/A	N/A

*Median instead of mean; N/A = not available.

doi:10.1371/journal.pone.0092316.t004

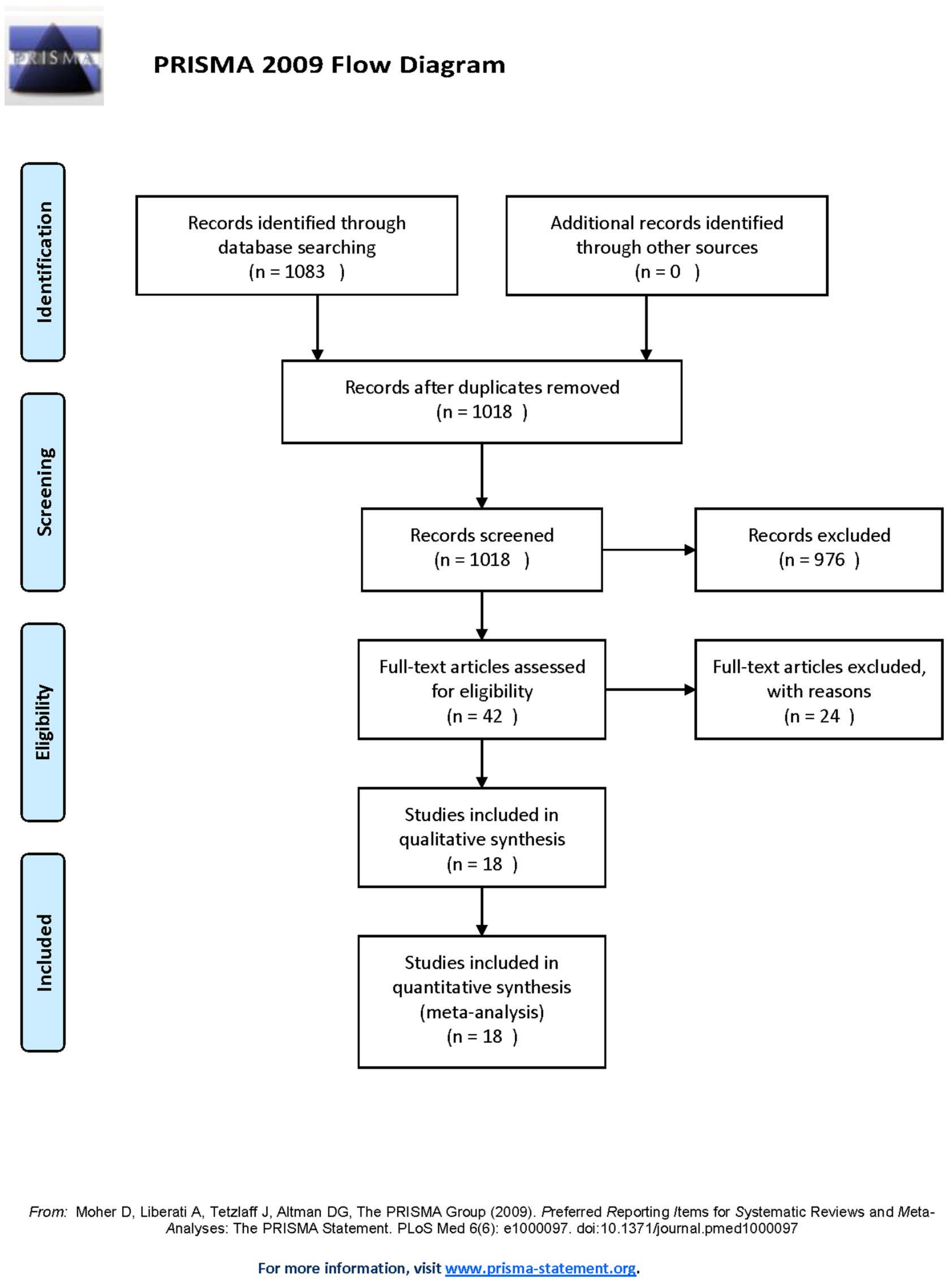

Figure 1. Flow diagram of study inclusion and exclusion.
doi:10.1371/journal.pone.0092316.g001

highly unstable condition (haemodynamic instability, heart failure, arrhythmias, resuscitation, and patient stress among others) that does not favor performance of PCI, and additional intervention is probably safer after the patient is stabilized; 2) the acute phase of STEMI is extremely prothrombotic and inflammatory which contributes to a higher risk for additional PCI; 3) diffuse coronary spasms (either due to endothelial dysfunction or due to catecholamine use) are frequently present in the acute phase of STEMI, and this may lead to overestimation of stenosis severity in non-culprit vessels; 4) decisions to perform non-culprit vessel PCI during the acute phase of STEMI are usually not supported by objective evidence for the presence of myocardial ischemia in regions supplied by these non-culprit vessels; 5) MV-PCI increases the radiation dose, contrast overload, and risk of contrast-induced nephropathy. Counter arguments include concerns that plaque instability may be present in large areas of the coronary tree rather

Study or Subgroup	culprit-PCI Events	culprit-PCI Total	MV-PCI Events	MV-PCI Total	Weight	Odds Ratio M-H, Random, 95% CI
**1.1.1 prospective**						
Di Mario 2004	0	17	1	52	1.6%	0.98 [0.04, 25.20]
Ochala 2004	0	44	0	48		Not estimable
Politi 2010	7	84	2	65	4.7%	2.86 [0.57, 14.27]
**Subtotal (95% CI)**		**145**		**165**	**6.3%**	**2.32 [0.55, 9.79]**
Total events	7		3			
Heterogeneity: Tau² = 0.00; Chi² = 0.34, df = 1 (P = 0.56); I² = 0%						
Test for overall effect: Z = 1.15 (P = 0.25)						
**1.1.2 retrospective**						
Abe 2013	12	220	11	54	8.6%	0.23 [0.09, 0.54]
Bauer 2011	72	2118	6	419	8.9%	2.42 [1.05, 5.61]
Cavender 2009	1321	25802	246	3134	13.1%	0.63 [0.55, 0.73]
Corpus 2004	23	354	5	26	7.4%	0.29 [0.10, 0.84]
Dziewierz 2010	42	707	9	70	9.4%	0.43 [0.20, 0.92]
Hannan 2010	10	503	17	503	9.2%	0.58 [0.26, 1.28]
Jensen 2013	4	820	25	354	7.4%	0.06 [0.02, 0.19]
Khattab 2008	2	45	1	28	2.5%	1.26 [0.11, 14.53]
Kornowski 2011	6	393	16	275	8.1%	0.25 [0.10, 0.65]
Qarawani 2008	1	25	4	95	2.9%	0.95 [0.10, 8.88]
Roe 2001	7	61	15	68	8.0%	0.46 [0.17, 1.21]
Varani 2008	8	156	12	147	8.3%	0.61 [0.24, 1.53]
**Subtotal (95% CI)**		**31204**		**5173**	**93.7%**	**0.45 [0.29, 0.71]**
Total events	1508		367			
Heterogeneity: Tau² = 0.36; Chi² = 39.33, df = 11 (P < 0.0001); I² = 72%						
Test for overall effect: Z = 3.50 (P = 0.0005)						
**Total (95% CI)**		**31349**		**5338**	**100.0%**	**0.50 [0.32, 0.77]**
Total events	1515		370			
Heterogeneity: Tau² = 0.37; Chi² = 43.09, df = 13 (P < 0.0001); I² = 70%						
Test for overall effect: Z = 3.14 (P = 0.002)						
Test for subgroup differences: Chi² = 4.52, df = 1 (P = 0.03), I² = 77.9%						

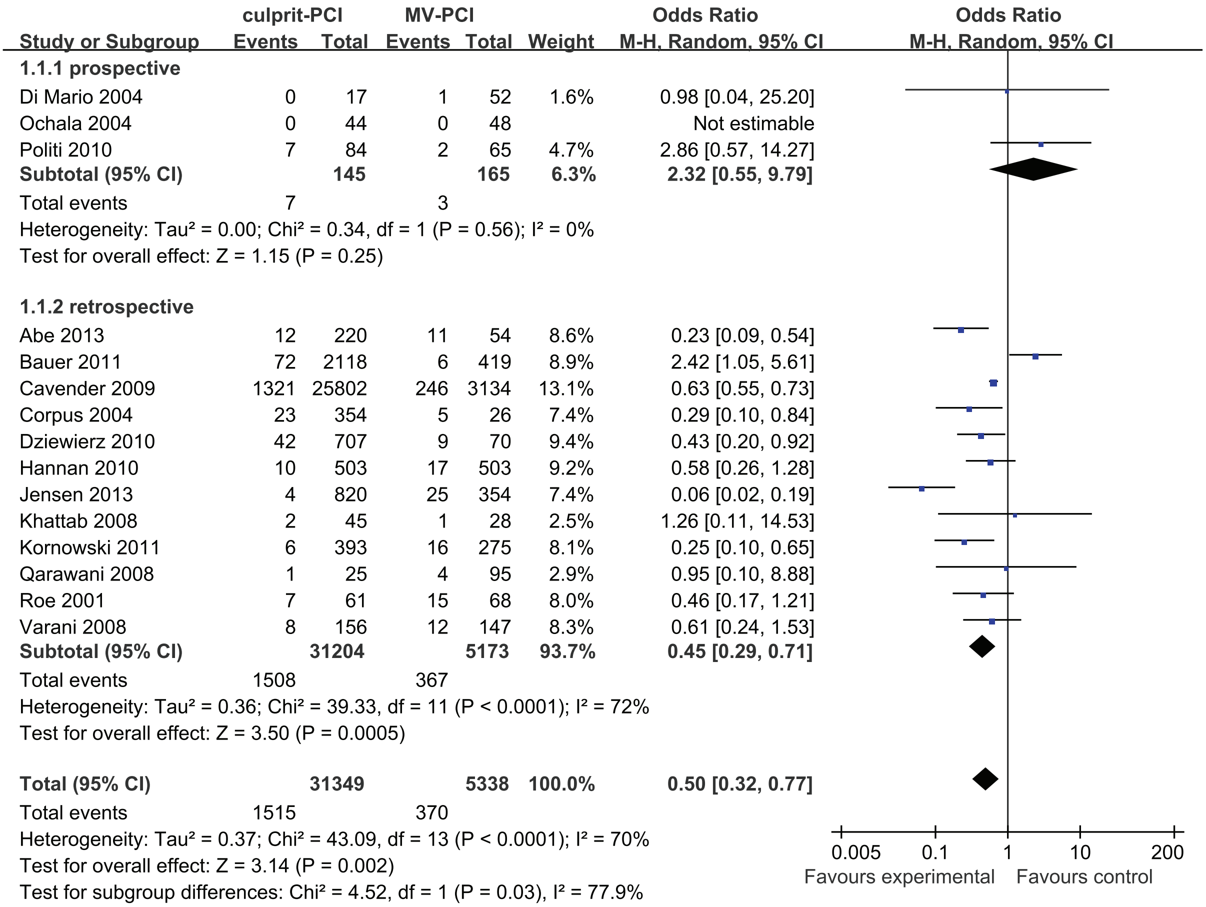

Figure 2. Culprit PCI Versus MV-PCI Short-Term Mortality.
doi:10.1371/journal.pone.0092316.g002

than limited to the culprit lesion. Consequently, MV-PCI might achieve complete revascularization by treating secondary unstable lesions and thereby shorten cumulative hospital stays and costs. Patients may also be more comfortable following treatment of all lesions during index hospitalization.

The report was written in accordance to the PRISMA-statement (Checklist S1).Our findings support the current guidelines which indicate that the non-culprit vessel should not be treated during the index procedure. Although analysis of only the four small scaled randomized trials has different even opposite results. It is notable that the largest single-blind, randomized study, called the Preventive Angioplasty in Acute Myocardial Infarction (PRAMI) trail [8], enrolled 465 patients at five centers in the United Kingdom, with 231 assigned to the culprit PCI group and 234 to the MV-PCI group. The recruitment was stopped early after a recommendation from the data and safety monitoring committee that was based on a highly significant difference between groups ($p<0.001$) in the incidence of the primary outcome favoring MV-PCI. The combined rate of cardiac death, nonfatal myocardial infarction, or refractory angina was reduced by 65%, and absolute risk reduction of 14 percentage points over 23 months. The findings also suggest that MV-PCI may lead to less ischemia testing after the index procedure. Another randomized trial enrolled only 214 patients, with 84 patients in the culprit PCI group, 65 in the MV-PCI group, and 65 in the staged PCI group [7]. This study showed a significant benefit for MV-PCI, compared to culprit PCI, for long-term major adverse cardiac events (MACE) after a mean follow-up of 2.5 years. The HEpacoat for cuLPrit or multivessel stenting for Acute Myocardial Infarction (HELP AMI) study [5] enrolled only 69 patients, with 17 patients in the culprit PCI group and 52 in the MV-PCI group. In this study, MV-PCI did not significantly increase in-hospital MACE (0 and 3.8% in culprit and MV-PCI groups, respectively, $p = 0.164$). Revascularization in the culprit PCI group at the 12 month follow-up was not statistically significant (35 vs 17%, $p = 0.247$). The trial's limitations included unequal randomization and use of heparin-coated stents which may be subject to bias.

A meta-analysis comparing culprit PCI, MV-PCI and staged PCI strategies found that MV-PCI was associated with highest mortality rates at both short- and long-term follow up, in which staged PCI strategy was defined as PCI confined to culprit vessel only, after which lesions in non-culprit vessel were treated during planned secondary procedures [24]. A proper analysis on the secondary endpoints was not possible because data were only available for a minority of the included studies. This meta-analysis included some patients with non-ST-segment elevation myocardial infarction (NSTEMI), and thus their included studies were different from ours. In addition, patients with STEMI or NSTEMI have different treating strategies.

Limitations

Only four studies were randomized. Consequently, the inclusion of nonrandomized studies introduces a potential selection bias, which means the benefit of culprit PCI shown in Table 5 may simply derive from selection bias towards patients with less severe

Study or Subgroup	culprit PCI Events	culprit PCI Total	MV-PCI Events	MV-PCI Total	Weight	Odds Ratio M-H, Random, 95% CI
**2.1.1 prospective**						
Di Mario 2004	0	17	0	52		Not estimable
Ochala 2004	0	44	0	48		Not estimable
Politi 2010	13	84	6	65	6.2%	1.80 [0.64, 5.03]
Wald 2013	16	231	12	234	7.9%	1.38 [0.64, 2.98]
**Subtotal (95% CI)**		**376**		**399**	**14.1%**	**1.52 [0.82, 2.81]**
Total events	29		18			
Heterogeneity: Tau² = 0.00; Chi² = 0.17, df = 1 (P = 0.68); I² = 0%						
Test for overall effect: Z = 1.32 (P = 0.19)						
**2.1.2 retrospective**						
Abe 2013	24	220	17	54	8.3%	0.27 [0.13, 0.54]
Corpus 2004	42	354	5	26	6.2%	0.57 [0.20, 1.58]
Dziewierz 2010	57	707	11	70	8.4%	0.47 [0.23, 0.95]
Hannan 2010	28	503	36	503	9.8%	0.76 [0.46, 1.27]
Jensen 2013	26	820	52	354	10.0%	0.19 [0.12, 0.31]
Khattab 2008	3	45	2	25	2.9%	0.82 [0.13, 5.28]
Kornowski 2011	9	393	25	275	7.8%	0.23 [0.11, 0.51]
Mohamad 2011	3	30	2	7	2.6%	0.28 [0.04, 2.11]
Qarawani 2008	2	25	9	95	3.6%	0.83 [0.17, 4.11]
Roe 2001	10	61	17	68	7.2%	0.59 [0.25, 1.41]
Toma 2010	111	1984	27	217	10.3%	0.42 [0.27, 0.65]
Varani 2008	18	152	24	142	8.7%	0.66 [0.34, 1.28]
**Subtotal (95% CI)**		**5294**		**1836**	**85.9%**	**0.42 [0.31, 0.59]**
Total events	333		227			
Heterogeneity: Tau² = 0.15; Chi² = 23.22, df = 11 (P = 0.02); I² = 53%						
Test for overall effect: Z = 5.17 (P < 0.00001)						
**Total (95% CI)**		**5670**		**2235**	**100.0%**	**0.52 [0.36, 0.74]**
Total events	362		245			
Heterogeneity: Tau² = 0.28; Chi² = 38.97, df = 13 (P = 0.0002); I² = 67%						
Test for overall effect: Z = 3.56 (P = 0.0004)						
Test for subgroup differences: Chi² = 12.81, df = 1 (P = 0.0003), I² = 92.2%						

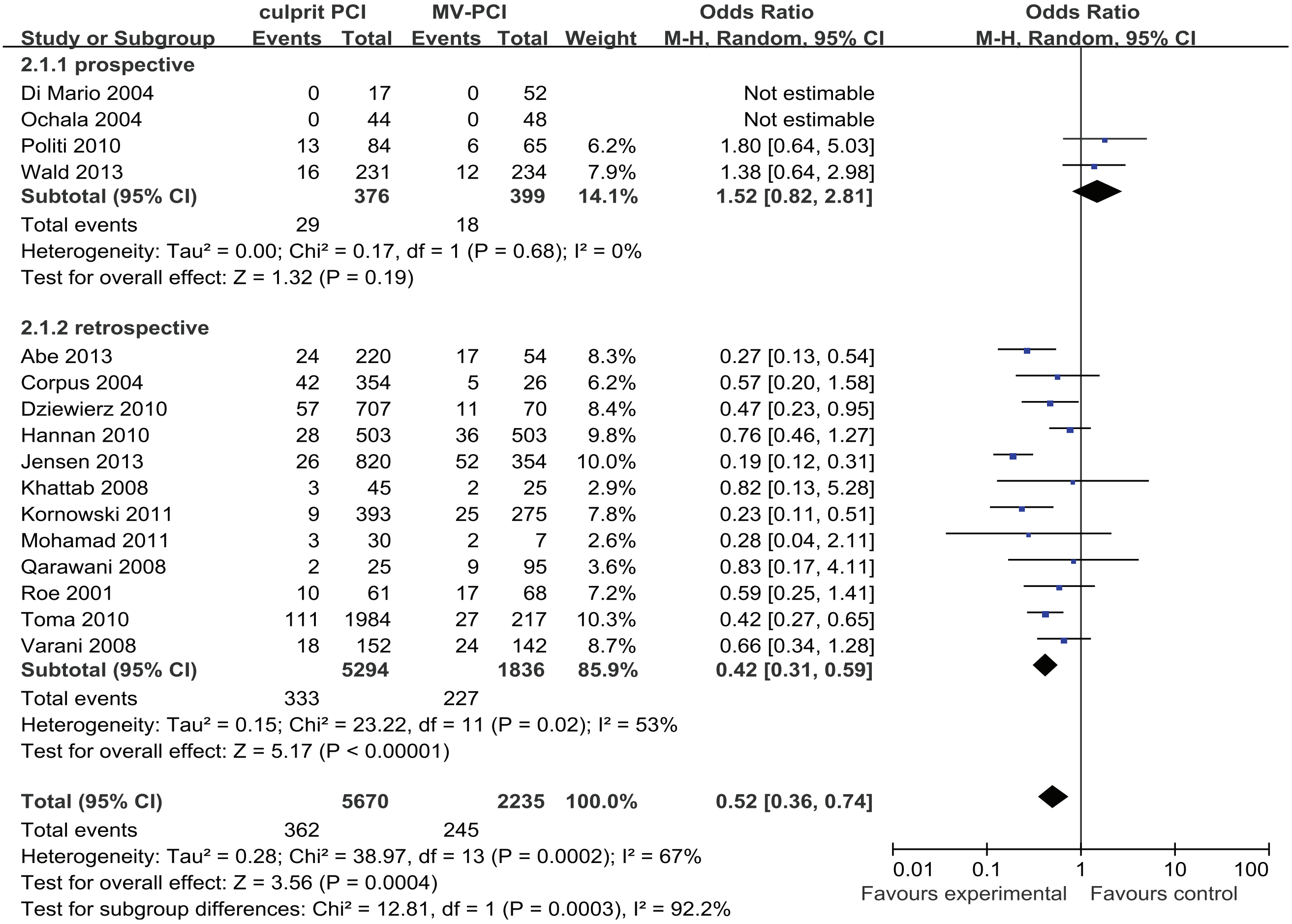

Figure 3. Culprit PCI Versus MV-PCI Long-Term Mortality.
doi:10.1371/journal.pone.0092316.g003

Study or Subgroup	culprit PCI Events	culprit PCI Total	MV-PCI Events	MV-PCI Total	Weight	Odds Ratio M-H, Fixed, 95% CI
**1.3.1 prospective**						
Politi 2010	3	84	1	65	0.7%	2.37 [0.24, 23.33]
**Subtotal (95% CI)**		**84**		**65**	**0.7%**	**2.37 [0.24, 23.33]**
Total events	3		1			
Heterogeneity: Not applicable						
Test for overall effect: Z = 0.74 (P = 0.46)						
**1.3.2 retrospective**						
Abe 2013	32	220	12	54	11.2%	0.60 [0.28, 1.25]
Cavender 2009	467	25802	72	3134	85.9%	0.78 [0.61, 1.01]
Qarawani 2008	1	25	8	95	2.2%	0.45 [0.05, 3.80]
**Subtotal (95% CI)**		**26047**		**3283**	**99.3%**	**0.76 [0.60, 0.96]**
Total events	500		92			
Heterogeneity: Chi² = 0.70, df = 2 (P = 0.71); I² = 0%						
Test for overall effect: Z = 2.34 (P = 0.02)						
**Total (95% CI)**		**26131**		**3348**	**100.0%**	**0.77 [0.61, 0.97]**
Total events	503		93			
Heterogeneity: Chi² = 1.64, df = 3 (P = 0.65); I² = 0%						
Test for overall effect: Z = 2.22 (P = 0.03)						
Test for subgroup differences: Chi² = 0.95, df = 1 (P = 0.33), I² = 0%						

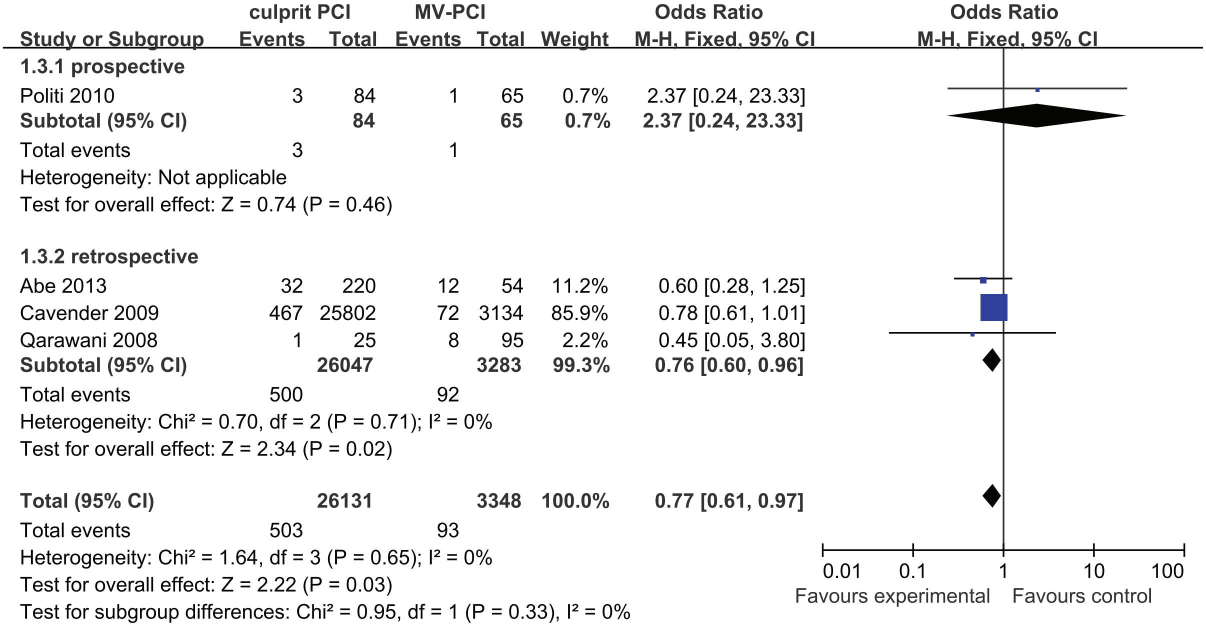

Figure 4. Culprit PCI Versus MV-PCI Renal Dysfunction.
doi:10.1371/journal.pone.0092316.g004

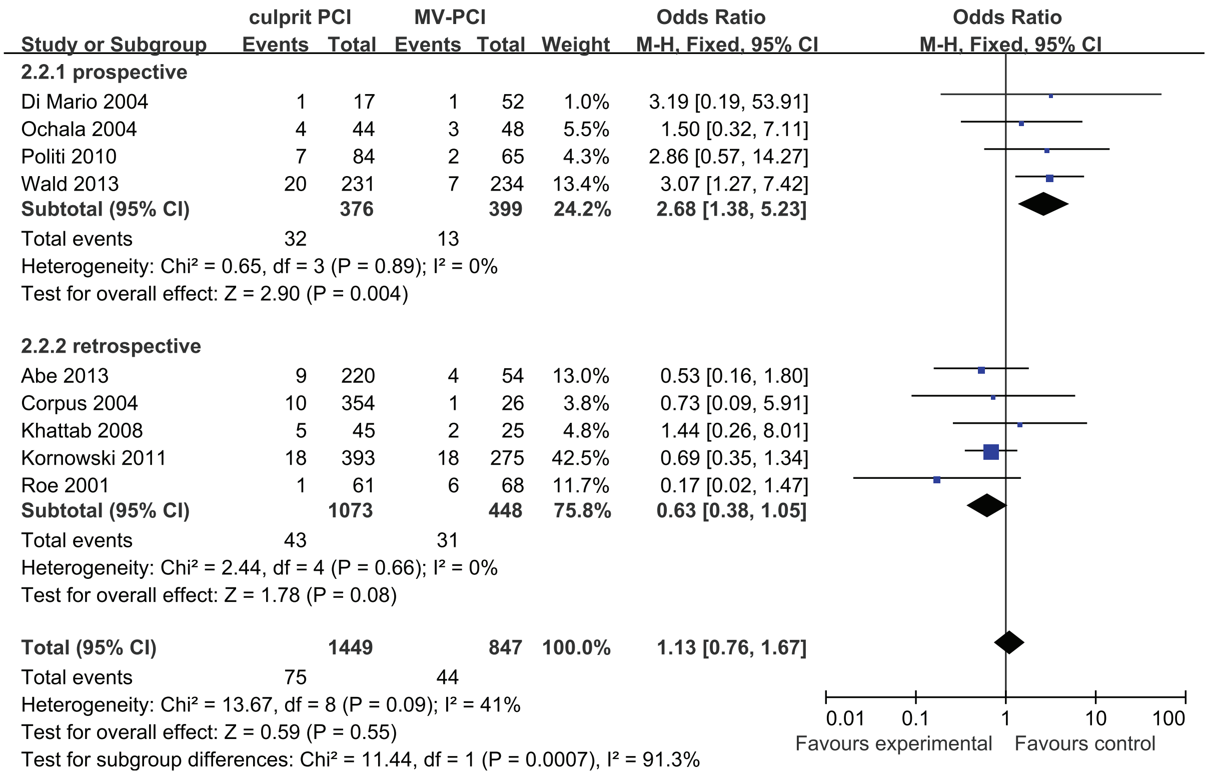

Figure 5. Culprit PCI Versus MV-PCI Long-Term Reinfarction.
doi:10.1371/journal.pone.0092316.g005

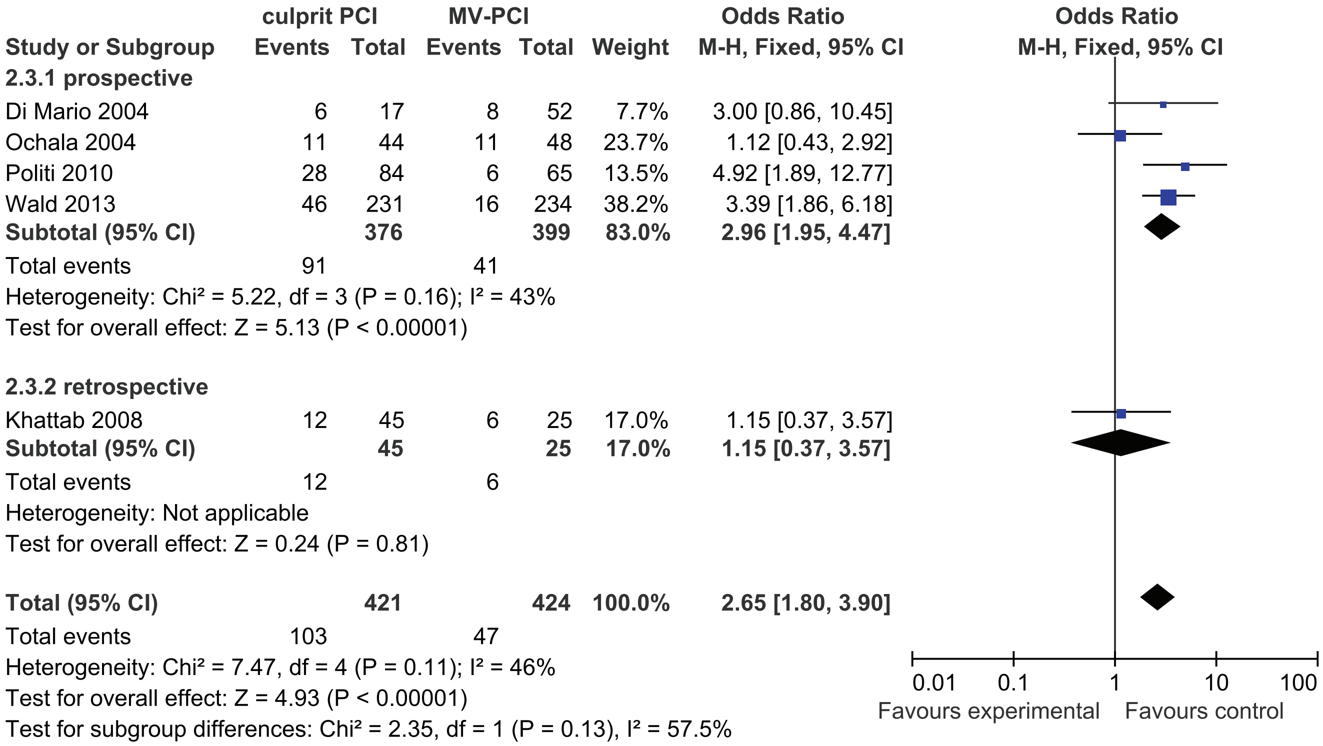

Figure 6. Culprit PCI Versus MV-PCI Long-Term Revascularization.
doi:10.1371/journal.pone.0092316.g006

Table 5. Subgroup Analysis of Randomized Trails Compared with Overall Analysis.

Endpoints	Preferred strategy	
	Randomized and nonrandomized trails	Randomized trails
Short-term mortality	Culprit PCI	Equal
Long-term mortality	Culprit PCI	Equal
Renal dysfunction	Culprit PCI	Equal
Reinfarction	Equal	MV-PCI
Revascularization	MV-PCI	MV-PCI

doi:10.1371/journal.pone.0092316.t005

or more stable coronary artery disease. There is the potential for ascertainment bias due to unequal follow-up.

Multiple combinations of angiographic and clinical findings, number of diseased vessels, location and type of occlusions, total chronic occlusions, Killip class, renal function, and other factors vary by individual. This introduces a level of complexity that is best addressed by individualized clinical decision-making.

Further, the operator's intent to perform culprit PCI or MV-PCI was not prospectively registered in a majority of the studies and may be influenced by important patient characteristics that we were unable to account for. Staged PCI was allowed for patients in culprit PCI group in some trials which may exaggerate the benefits of culprit PCI. As with many meta-analyses, we did not adjust our analyses for baseline confounders or unmeasured confounders, due to the lack of data in each trial.

Conclusions

This meta-analysis was based primarily on data derived from nonrandomized studies. It is suggested that culprit PCI is better than MV-PCI procedure in patients with STEMI and MVD. Large-scale randomized trials are urgently needed to further evaluate different revascularization procedures for patients with STEMI and MVD.

Supporting Information

Checklist S1 PRISMA Checklist of items to include when reporting a systematic review or meta-analysis. (DOC)

Author Contributions

Conceived and designed the experiments: DFZ XTS SZL. Performed the experiments: DFZ FX MZ WL SY. Analyzed the data: DFZ FX MZ WL SY. Wrote the paper: DFZ XTS FY.

References

1. Sorajja P, Gersh BJ, Cox DA, McLaughlin MG, Zimetbaum P, et al. (2007) Impact of multivessel disease on reperfusion success and clinical outcomes in patients undergoing primary percutaneous coronary intervention for acute myocardial infarction. Eur Heart J 28: 1709–1716.
2. Steg PG, James SK, Atar D, Badano LP, Blomstrom-Lundqvist C, et al. (2012) ESC Guidelines for the management of acute myocardial infarction in patients presenting with ST-segment elevation. Eur Heart J 33: 2569–2619.
3. Levine GN, Bates ER, Blankenship JC, Bailey SR, Bittl JA, et al. (2011) 2011 ACCF/AHA/SCAI Guideline for Percutaneous Coronary Intervention. A report of the American College of Cardiology Foundation/American Heart Association Task Force on Practice Guidelines and the Society for Cardiovascular Angiography and Interventions. J Am Coll Cardiol 58: e44–e122.
4. Stroup DF, Berlin JA, Morton SC, Olkin I, Williamson GD, et al. (2000) Meta-analysis of observational studies in epidemiology: a proposal for reporting. Meta-analysis Of Observational Studies in Epidemiology (MOOSE) group. JAMA 283: 2008–2012.
5. Di Mario C, Mara S, Flavio A, Imad S, Antonio M, et al. (2004) Single vs multivessel treatment during primary angioplasty: results of the multicentre randomised HEpacoat for cuLPrit or multivessel stenting for Acute Myocardial Infarction (HELP AMI) Study. Int J Cardiovasc Intervent 6: 128–133.
6. Ochala A, Smolka GA, Wojakowski W, Dudek D, Dziewierz A, et al. (2004) The function of the left ventricle after complete multivessel one-stage percutaneous coronary intervention in patients with acute myocardial infarction. J Invasive Cardiol 16: 699–702.
7. Politi L, Sgura F, Rossi R, Monopoli D, Guerri E, et al. (2010) A randomised trial of target-vessel versus multi-vessel revascularisation in ST-elevation myocardial infarction: major adverse cardiac events during long-term follow-up. Heart 96: 662–667.
8. Wald DS, Morris JK, Wald NJ, Chase AJ, Edwards RJ, et al. (2013) Randomized trial of preventive angioplasty in myocardial infarction. N Engl J Med 369: 1115–1123.
9. Abe D, Sato A, Hoshi T, Takeyasu N, Misaki M, et al. (2013) Initial culprit-only versus initial multivessel percutaneous coronary intervention in patients with ST-segment elevation myocardial infarction: results from the Ibaraki Cardiovascular Assessment Study registry. Heart Vessels.
10. Bauer T, Zeymer U, Hochadel M, Mollmann H, Weidinger F, et al. (2013) Prima-vista multi-vessel percutaneous coronary intervention in haemodynamically stable patients with acute coronary syndromes: analysis of over 4.400 patients in the EHS-PCI registry. Int J Cardiol 166: 596–600.
11. Cavender MA, Milford-Beland S, Roe MT, Peterson ED, Weintraub WS, et al. (2009) Prevalence, predictors, and in-hospital outcomes of non-infarct artery intervention during primary percutaneous coronary intervention for ST-segment elevation myocardial infarction (from the National Cardiovascular Data Registry). Am J Cardiol 104: 507–513.
12. Corpus RA, House JA, Marso SP, Grantham JA, Huber KJ, et al. (2004) Multivessel percutaneous coronary intervention in patients with multivessel disease and acute myocardial infarction. Am Heart J 148: 493–500.
13. Dziewierz A, Siudak Z, Rakowski T, Zasada W, Dubiel JS, et al. (2010) Impact of multivessel coronary artery disease and noninfarct-related artery revascularization on outcome of patients with ST-elevation myocardial infarction transferred for primary percutaneous coronary intervention (from the EURO-TRANSFER Registry). Am J Cardiol 106: 342–347.
14. Hannan EL, Samadashvili Z, Walford G, Holmes DJ, Jacobs AK, et al. (2010) Culprit vessel percutaneous coronary intervention versus multivessel and staged percutaneous coronary intervention for ST-segment elevation myocardial infarction patients with multivessel disease. JACC Cardiovasc Interv 3: 22–31.
15. Jensen LO, Thayssen P, Farkas DK, Hougaard M, Terkelsen CJ, et al. (2012) Culprit only or multivessel percutaneous coronary interventions in patients with ST-segment elevation myocardial infarction and multivessel disease. EuroIntervention 8: 456–464.
16. Khattab AA, Abdel-Wahab M, Rother C, Liska B, Toelg R, et al. (2008) Multivessel stenting during primary percutaneous coronary intervention for acute myocardial infarction. A single-center experience. Clin Res Cardiol 97: 32–38.
17. Kornowski R, Mehran R, Dangas G, Nikolsky E, Assali A, et al. (2011) Prognostic impact of staged versus "one-time" multivessel percutaneous intervention in acute myocardial infarction: analysis from the HORIZONS-AMI (harmonizing outcomes with revascularization and stents in acute myocardial infarction) trial. J Am Coll Cardiol 58: 704–711.
18. Mohamad T, Bernal JM, Kondur A, Hari P, Nelson K, et al. (2011) Coronary revascularization strategy for ST elevation myocardial infarction with multivessel disease: experience and results at 1-year follow-up. Am J Ther 18: 92–100.
19. Qarawani D, Nahir M, Abboud M, Hazanov Y, Hasin Y (2008) Culprit only versus complete coronary revascularization during primary PCI. Int J Cardiol 123: 288–292.

20. Roe MT, Cura FA, Joski PS, Garcia E, Guetta V, et al. (2001) Initial experience with multivessel percutaneous coronary intervention during mechanical reperfusion for acute myocardial infarction. Am J Cardiol 88: 170–173, A6.
21. Toma M, Buller CE, Westerhout CM, Fu Y, O'Neill WW, et al. (2010) Non-culprit coronary artery percutaneous coronary intervention during acute ST-segment elevation myocardial infarction: insights from the APEX-AMI trial. Eur Heart J 31: 1701–1707.
22. Varani E, Balducelli M, Aquilina M, Vecchi G, Hussien MN, et al. (2008) Single or multivessel percutaneous coronary intervention in ST-elevation myocardial infarction patients. Catheter Cardiovasc Interv 72: 927–933.
23. Widimsky P, Holmes DJ (2011) How to treat patients with ST-elevation acute myocardial infarction and multi-vessel disease? Eur Heart J 32: 396–403.
24. Vlaar PJ, Mahmoud KD, Holmes DJ, van Valkenhoef G, Hillege HL, et al. (2011) Culprit vessel only versus multivessel and staged percutaneous coronary intervention for multivessel disease in patients presenting with ST-segment elevation myocardial infarction: a pairwise and network meta-analysis. J Am Coll Cardiol 58: 692–703.
